# Boosted Cross-Linking and Characterization of High-Performing Self-Assembling Peptides

**DOI:** 10.3390/nano12030320

**Published:** 2022-01-19

**Authors:** Maria Gessica Ciulla, Raffaele Pugliese, Fabrizio Gelain

**Affiliations:** 1 Institute for Stem-Cell Biology, Regenerative Medicine and Innovative Therapies, IRCCS Casa Sollievo della Sofferenza, 71013 San Giovanni Rotondo, Italy; mg.ciulla@operapadrepio.it (M.G.C.); raffaele.pugliese@nemolab.it (R.P.); 2 NeMo Lab, ASST Grande Ospedale Metropolitano Niguarda, 20162 Milan, Italy; 3 Center for Nanomedicine and Tissue Engineering (CNTE), ASST Grande Ospedale Metropolitano Niguarda, 20162 Milan, Italy

**Keywords:** self-assembling peptides, nanofibers, Sulfo-SMCC, cross-linking, biomaterials, rheology

## Abstract

Tissue engineering (TE) strategies require the design and characterization of novel biomaterials capable of mimicking the physiological microenvironments of the tissues to be regenerated. As such, implantable materials should be biomimetic, nanostructured and with mechanical properties approximating those of the target organ/tissue. Self-assembling peptides (SAPs) are biomimetic nanomaterials that can be readily synthesized and customized to match the requirements of some TE applications, but the weak interactions involved in the self-assembling phenomenon make them soft hydrogels unsuited for the regeneration of medium-to-hard tissues. In this work, we moved significant steps forward in the field of chemical cross-linked SAPs towards the goal of stiff peptidic materials suited for the regeneration of several tissues. Novel SAPs were designed and characterized to boost the 4-(*N-*Maleimidomethyl) cyclohexane-1-carboxylic acid 3-sulpho-*N*-hydroxysuccinimide ester (Sulfo-SMCC) mediated cross-linking reaction, where they reached G′ values of ~500 kPa. An additional orthogonal cross-linking was also effective and allowed to top remarkable G′ values of 840 kPa. We demonstrated that cross-linking fastened the pre-existing self-aggregated nanostructures, and at the same time, a strong presence of ß-structures is necessary for an effective cross-linking of (LKLK)_3_-based SAPs. Combining strong SAP design and orthogonal cross-linking reactions, we brought SAP stiffness closer to the MPa threshold, and as such, we opened the door of the regeneration of skin, muscle and lung to biomimetic SAP technology.

## 1. Introduction

**Figure 1:**
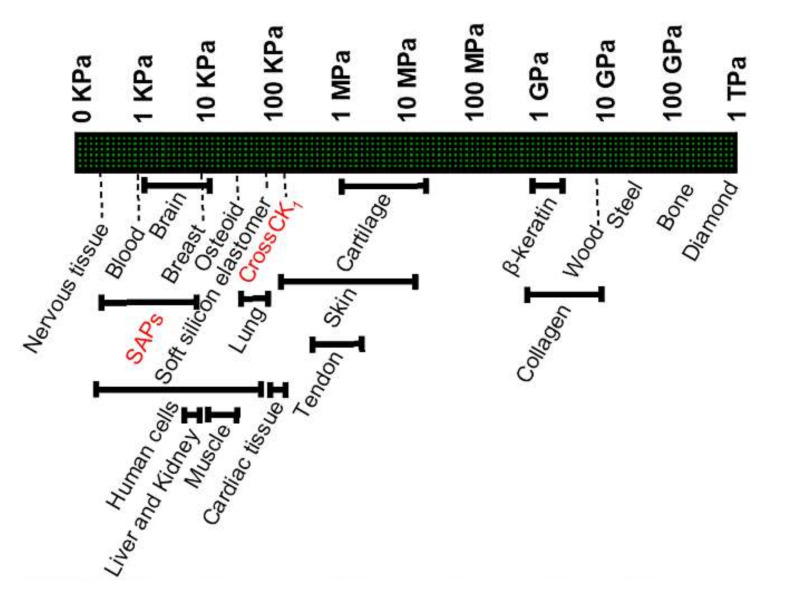
Elastic moduli of different tissues and materials: standard plain SAPs lay in the range of soft hydrogels. Recently published works on cross-linked SAPs showed highest G′ values of approximately 170 kPa, hence the typical stiffness of various tissues is still out of reach [20].

The development of new nanomaterials with enhanced structural and mechanical properties is crucial to improve the outcomes of tissue engineering strategies, and for example, tackle the regeneration of either extra-soft or hard tissues. Molecular self-assembly is a powerful approach for fabricating novel supramolecular architectures [1]. This phenomenon, ubiquitous in biological systems, takes place thanks to a delicate balance between effects of solvation and different non-covalent interactions such as H-bonding, π-π type or generic hydrophobic interactions, van der Waals type interactions [2]. Self-assembly and self-organization, as dominant processes in the chemistry of living biological system [3], organize small molecules into ordered structures on multiple length scales. In the recent years, researchers showed that peptides are among the best candidates for the formation of supramolecular organized scaffolds [4]. Indeed, peptide-based materials have great potential for new therapies in regenerative medicine [5] thanks to their inherent molecular recognition capabilities, biocompatibility, and negligible toxicity in biological fluids. Self-assembling peptides (SAPs) are synthetic bioactive nanomaterials used to obtain a variety of nanofibrous structures and they constitute one of the most important classes of synthetic self-assembling hydrogels [6]. SAP scaffolds are mimicry of the extracellular matrix (ECM), provide biochemical cues triggering biological response (e.g., cell adhesion, proliferation and differentiation), and thanks to their intrinsic versatility, they can be used for studying Alzheimer’s disease [7], the regeneration of spinal cord and brain injuries [8,9], in cartilage tissue engineering [10], in cell and organoid culturing [11], drug discovery [12], bioimaging and drug delivery [13]. Nonetheless, SAPs usually give yield to soft fragile hydrogels, suited for soft tissue regeneration or as fillers [14] (Figure 1): that is why recent efforts have been focused on enhancing the mechanical properties of SAP hydrogels to match those of the tissues to be regenerated [15,16,17,18,19].

**Figure 2:**
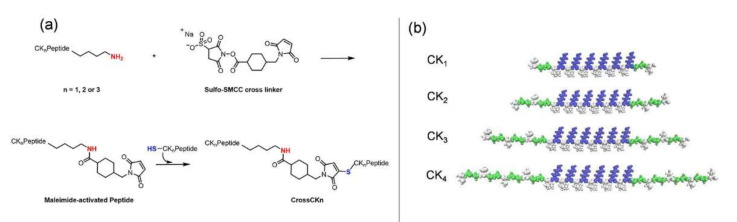
(**a**) Scheme of reaction mechanism of Sulfo-SMCC cross-linking as suggested by seller Termofisher (link at: https://www.thermofisher.com/order/catalog/product/22322 accessed on 14 January 2022) [26]. The preparation of crossCKs peptides undergoes a two-steps conjiugation with a molar excess of the cross-linker. (**b**) VDW representation of CK_1_, CK_2_, CK_3_ and CK_4_ molecules. Coloring method follows residue type.

While multi-functionalization (i.e., biomimetic property) and their modifications can be considered as accomplished milestones for LDLK- or RADA-based SAPs [21]], tuning their stiffness across various orders of magnitude, in order to match that one of various living tissues (Figure 1) [20], is still an open quest. Cross-linking modifications might assist to enhance mechanical properties of a material and chemical cross-linkers are used to bind peptides and proteins for numerous applications [22]. Cross-linkers have been shown to improve scaffolds performances and thus have attracted great interest [23]. Various cross-linking methods are available, both physical and chemical and they can be classified according to their chemical specificity; to the length of the cross-bridge formed; to the homobifunctionality or heterobifunctionality of the cross-linker; to the type of reaction (e.g., chemical or photo-chemical); to the presence of a cleaved bond in the reaction; and to the amenability for further radiolabeling [24]. Heterobifunctional cross-linkers have two different groups allowing for sequential conjugations: among them, some cross-linkers contain an NHS ester with a second functional group capable of binding to a sulfhydryl. A common sulfhydryl-reactive group is the maleimide moiety, that reacts with cysteine residues in slightly acidic-to-neutral conditions [25]. Sulfo-SMCC is an NHS-ester end of a heterobifunctional cross-linker reactive against primary amines of lysine, making a maleimide-activated peptide. At the same time, sulfhydryl groups react with the maleimide groups, thus yielding to a “molecular bridge” (Figure 2a) [26].

In our previous study [27], we demonstrated the increased mechanical properties of a promising SAP, CK_1_ (the top one in Figure 2b), after Sulfo-SMCC cross-linking reaction, wherein the peptide was covalently cross-linked to make a stronger gel [6]. Despite the significant advance, a good percentage of unreacted primary amines and sulfhydryl groups was left at the end of the reaction, demonstrating the chance for further improvements of the proposed approach. Here, three new SAPs belonging to the CK family (Figure 2b), CK_2_, CK_3_ and CK_4_, were designed, synthesized and characterized to explore their mechanical properties, evaluate their self-assembling propensity, their reactivity to the cross-linking reaction and, as such, their feasibility as high-performing peptidic scaffolds. Furthermore, we reported the combined double cross-linking of SAPs via Sulfo-SMCC and Genipin (GP) (a natural cross-linker), where peptides were firstly covalently linked with Sulfo-SMCC, and consequently treated with GP [28]. The final aim was to saturate all available binding sites in peptide sequences by boosting this cross-linking approach to the maximum limit, and as such, to assess its potential in terms of enhanced scaffold stiffness. The designed crossCK_n_ and crossCK_n_-GP were characterized and investigated by measurements and experimental assays, testifying the increased mechanical toughness of the biomaterials after cross-linking reaction, but also the limit to the proposed strategy in terms of SAP functionalization.

In this scenario, crossCK_n_ materials may represent new versatile synthons in the SAPs family, which can be additionally double-cross-linked, orthogonally functionalized, and as such, can be considered steppingstones for novel applications.

## 2. Materials and Methods

### 2.1. Materials

All chemicals were purchased from Merck (Merck Millipore, Darmstadt, Germany), Sigma Aldrich (Sigma Aldrich Chemie GmbH, München, Germany) and VWR (Radnor, PA, USA) in highest quality commercially available. The solvents are capable of RP (Reverse Phase). Fmoc-protected amino acids were obtained from CEM (Matthews, NC, USA) and used as received without further purification. Sulfo-SMCC was purchased from Thermo Fisher (Fischer Scientific GmbH, Schwerte, Germany). The structure of intermediates and the final products were evaluated through exact MS and IR. LC-MS spectra were recorded via single quadrupole mass detection (Waters LC-MS Alliance 3100, Waters Corp., Milford, CT, USA) using a nebulizing nitrogen gas at 400 L/min and a temperature of 250 °C, cone flow 40 mL/min, capillary 3.5 Kvolts and cone voltage 60 V; or with a Waters ACQUITY UPLC system (Waters Corp., Milford, CT, USA) coupled with a Waters Xevo G2-XS QTof Mass Spectrometer in a positive mode using a temperature of 150 °C, cone flow 20 mL/min, capillary 3 kVolts and cone voltage 20 V.

### 2.2. General Procedure for Peptides Synthesis

Synthesis was carried out via microwave solid-phase Fmoc-based chemistry using a CEM Liberty Blue system (CEM Corp., Matthews, NC, Canada) on a 0.25 mmol scale in excess of a 0.2 M amino acid solution (in DMF) with 1 M DIC (in DMF) as an activator and 1 M Oxyma (in DMF) as an activator base. A 0.56 mmol/g Rink Amide resin was used for the synthesis of CK_1_ and CK_2_, while a 0.19 mmol/g Rink Amide resin was used for CK_3_ and CK_4_. Fmoc-protected amino acids were dissolved at 0.2 M in DMF and the deprotection solution used for the removal of Fmoc groups was a 10% *v*/*v* of piperazine in 9:1 NMP/EtOH solution. The N-terminal was acetylated using a 20% *v*/*v* solution of Ac_2_O in DMF. The peptide resin was washed three times with CH_2_Cl_2_ immediately after synthesis. Cleavage was then performed in 10 mL of a freshly prepared TFA:H_2_O:DODt:TIS (92.5:2.5:2.5:2.5) solution for 30 min utilizing a CEM RAZOR System with a temperature set at 38 °C. The resin was then filtered and the TFA solution added to ice cold diethyl ether for precipitation and then lyophilized (Labconco, Kansas City, MO, USA). Purification of the crude material was carried out using a Waters binary HPLC (>96%) on a Restek^TM^ (Restek Corp., Bellefonte, PA, USA) Prep C18 column. The mobile phase consisted of a gradient of acetonitrile with 0.1% TFA and H_2_O with 0.1% TFA over 30 min. After semi-preparative HPLC purification, the isolated product was used to the final concentrations (see analytical section in Supplementary Materials).

### 2.3. Cross-Linking Reaction

A solution (1:1 H_2_O:DPBS) of Sulfo-SMCC was slowly added to a previously dissolved 20 mM of all CK_s_ (2 equiv., 4 equiv., 6 equiv. and 8 equiv. for, respectively, CK_1_, CK_2_, CK_3_ or CK_4_. After addition, the reaction was stirred overnight at room temperature. Then the unreacted Sulfo-SMCC was removed with a vacuum pipette. Finally, the cross-linked material was washed with DPBS.

For double cross-linking, a solution of 170 mM of GP dissolved in 95:5 H_2_O:EtOH was added to crossCKs, and the reaction solution was then incubated at 37 °C. After 24 h, the crossCKn-GP was washed five times with DPBS to remove the unreacted GP.

### 2.4. Characterization of SAPs and Cross-SAPs

#### 2.4.1. Rheological Characterization

Rheological characterization of the prepared materials at different concentrations (see Results for details) was performed by the rotational AR-2000 ex rheometer (TA Instruments, Waters Corp., Milford, CT, USA). A cone–plate configuration with an acrylic diameter of 20 mm and a truncation of 34 μm and angle 1% was used. Exclusively for the double cross-linking with GP a plate-plate configuration with a diameter of 20 mm was used. The temperature for rheological tests was set to 25° C. A time sweep experiment was performed at 1 Hz constant angular frequency, followed by a frequency sweep test (1–1000 Hz) at the constant strain of 1% in the linear viscoelastic range experiments and as a function angular frequency. Stress/strain amplitude was performed from 0.01% to 1000%. G′ (storage modulus) and G″ (loss modulus) representing, respectively, the solid-like and the liquid-like behavior of the materials recorded. All measurements were performed in triplicate and curves were processed with OriginPro 2019 (OriginLab Corporation, Northampton, MA, USA). All results regarding the loss modulus G′′ experiments were reported in the Supplementary Materials.

#### 2.4.2. Fourier-Transform Infrared Spectroscopy in Attenuated Total Reflection Mode (ATR-FTIR) Analysis

Attenuated total reflection spectra were collected using a Perkin-Elmer Spectrum Two IR spectrometer (PerkinElmer Ltd., Beaconsfield, United Kingdom) equipped with a Perkin-Elmer single-reflection diamond ATR. The spectra of the samples were measured in the range of 400 to 4000 cm^−1^ at room temperature. Particular attention was paid in the infrared spectra in the Amide A, Amide I, Amide II band regions. The spectra were analyzed using OriginPro 2019 (OriginLab Corporation, Northampton, MA, USA). Firstly, baseline correction was performed, and hidden peaks were detected using a second derivative method followed by smoothing with the 7–9 point Savitsky–Golay function with a polynomial order of 2. Peak fitting/deconvolution was then performed using the Voigt function from OriginPro which is the convolution of a Gaussian function and a Lorentzian function [29].

#### 2.4.3. Thioflavin-T (ThT) Binding Assay

The benzothiol dye ThT was used to detect the β-sheets in CKs and cross-CKs: ThT links β-sheets [30] in a fibril-ligand interaction between dye and rows of repetitive amino acid patterns running perpendicular to the strands that represent a ubiquitous structural feature of fibril-like surfaces. A solution of 20 mM CK_n_ in milli-Q water (pH 7.4) was mixed with a ThT working solution (ThT/CK_n_ 0.5:1) and was stirred for 2 min [31]. ThT fluorescence measurements were recorded using an Infinite M200 Pro plate reader (Tecan, Mennedorf, Switzerland) with excitation at 440 nm and emission 482 nm. All measurements were carried out in 1 cm path length micro-fluorescence cells.

#### 2.4.4. Quantification of Amino Groups on the Uncross-Linked via 2,4,6-Trinitrobenzene Sulfonic Acid (TNBSA) Assay

This method is used to indirectly determine the number of the remaining unmodified lysine residues after the cross-reaction [32]. TNBSA in 0.1 M aqueous buffer at pH 8.5 was added to a 20 mM peptide solution in H_2_O and the reaction was stirred for 2 h at 37 °C. The reaction mixture was quenched with a solution of 1 N HCl. Reaction with primary amines was observed by producing a chromogenic yellow colored derivative and the absorbance was recorded in the ultraviolet light at 335 nm using an Infinite M200 Pro plate reader (Tecan). Absorbance curve resulting from TNBSA assay was monitored at 0, 2, 4, 6, 8 and 24 h. All measurements were carried out in triplicate in 1 cm path length micro-fluorescence cells and processed with OriginPro using Boltzmann fitting.

#### 2.4.5. Quantification of Free Thiols Using Ellman’s Reagent

In a 1 cm sample cuvette, 100 µL of freshly prepared Ellman’s solution [33] were added to a 20 mM peptide aqueous solution. After mixing for 15 min, free thiols percentage was recorded by reading the absorbance in an Infinite M200 Pro plate reader (Tecan) at 412 nm. The calculations from molar absorbance of TNB anion giving E_412_ was TNB^−2^ = 1.36 × 10^4^ cm^−1^M^−1^. Absorbance curve resulting from Ellman’s assay was monitored at 0, 2, 4, 6, 8 and 24 h. All measurements were carried out in 1 cm path length micro-fluorescence cells and processed with OriginPro using Boltzmann fitting.

#### 2.4.6. Atomic Force Microscopy (AFM) Analysis

Images captured were obtained in tapping mode using a Multimode Nanoscope V (Digital Instruments, Veeco, Plainview, NY, USA), with a single cantilever beam probe (Veeco RFESP MPP-21100-10, cantilever f0, 76–90 kHz). When necessary, data sets were subjected to a first-order flattening. Measured fiber dimensions were corrected because of the convolution effect arising from the finite size of the AFM tip. The observed nanofiber heights being far lower than the tip radius and the observed widths could be corrected with the formula [34]:

$$\Delta x=\sqrt{2\left[h\left(2rt-h\right)\right]}$$

where Δ*x* is the width broadening effect, *h* is the nanofiber height, and *rt* is the tip radius. AFM images were traced using FiberApp software [35].

## 3. Results and Discussion

### 3.1. Design of High-Performing Cross-Linked SAPs

In the search of bioinspired synthons for biomaterials design, the sol-to-gel transition of SAPs was combined with a strong cross-linker such as Sulfo-SMCC. We designed novel CK_n_ taking inspiration from our previously reported results, where the peptide sequence called CK, now dubbed CK_1_, showed increased stiffness and tailorability once treated with Sulfo-SMCC, without undermining its self-assembling propensity. We focused our efforts on modulating the length of the peptide chains and on the optimization of the concentration of Sulfo-SMCC in reference to the number of sulfhydryl groups introduced. The chosen self-assembly backbone is LKLK12 (Ac-LKLKLKLKLKLK-CONH_2_) [36], that self-assembles and triggers a sol-to-gel transition upon exposure to a basic pH-shift. LKLK12 backbone, positively charged due to Lys alternated pattern, was used in our previous studies32 on complementary co-assembling peptide (CAP) hydrogels based on mutual-attraction and self-repulsion of positive and negative modules. Functionalization of LKLK12 backbone was designed by keeping in mind the need for a glycine-spacer [37], usually present in standard SAPs, to preserve both the β-structuring of the backbone and the exposure of the added functional/active motifs. Although a good stiffness value (G′ = 170 kPa) with CK_1_ was achieved in our previous work, this was still far from covering the stiffnesses required in tissue engineering applications other than those of soft tissues [38]. Furthermore, the detected presence of unreacted amine groups at the end the cross-linking reaction pointed at additional room for improvement of the proposed strategy, e.g., increasing the number of sulfhydryl active sites on the SAP termini while preserving the same self-assembling backbone. Thus, three novel peptides, CK_2_, CK_3_ and CK_4_, belonging to the CK family were synthesized and evaluated to improve the performances of CK_1_.

All CK_n_ are symmetrical peptides against to the central backbone: they incorporate increasing numbers of repeated units of (Cys-Gly-Gly)_n_ and (Gly-Gly-Cys)_n_ at both ends, with *n* = 1, 2, 3, 4, respectively for CK_1_, CK_2_, CK_3_ and CK_4_ (see Figure 1 above) (see Supplementary Materials).

### 3.2. Rheology

**Figure 3:**
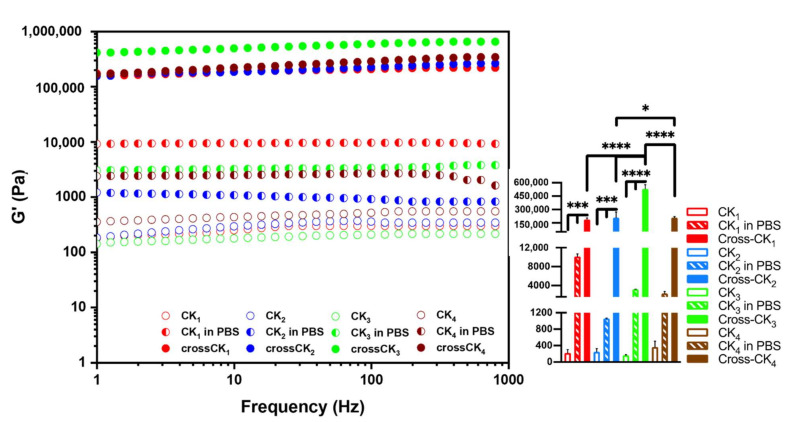
Rheological tests. The mean values of elastic moduli, G′, calculated after Sulfo-SMCC cross-link reaction (15 h) via a frequency-sweep test. All experiments were performed in triplicate. CrossCK_3_ tops an average G′ value around 532 kPa. Statistical analysis was performed by Ordinary One-way ANOVA. * *p* < 0.01, *** *p* = 0.001 and **** *p* < 0.0001 indicate the significance regarding stiffness of materials analyzed.

Rheological properties are indicators of the strength of a fibrillar network, and its stiffness tunability well-correlates with the range of potential applications in biotechnology. To investigate the mechanical properties of SAP scaffolds, we evaluated the storage (G′) and loss (G″) moduli as function of angular frequency [39] (Figure 3).

The initial peptide concentration was 20 mM, that is the maximum concentration warranting appropriate solubility for this kind of SAPs. After self-assembling (i.e., addition of PBS), all CK_n_ showed G′ >> G″, confirming their solid-like behavior (Figure 1 and Figure S1, Tables S1 and S2). In general, the cross-linking reaction was expected to increase the stiffness of all tested SAPs. Furthermore, all the crossCKs storage moduli were >100 kPa, ranking their stiffness between those of platelets and cartilage.

Best results were achieved with crossCK_3_ with a G′ value of (532 ± 44) kPa. Our data confirmed that the number of Cys is the major factor responsible for the increase of storage moduli: indeed, G′ values are higher in the ascending order (CK_3_ > CK_2_ > CK_1_). Nonetheless, we faced a chain length limit to this approach, being both G′_CK4_ and G′_crossCK4_ values lower than, respectively G′_CK3_ and G′_crossCK3_. Similarly to RADA16-I, RADA16-II, EAK16, and KLDL12 [40], which are 12–30 residues in length, the formation of supramolecular cross-β structures comes from a subtle balance between hydrophobic and electrostatic interactions [41], responsible for their self-assembly propensity [42]. We hypothesized that overlong functional motifs may have influenced the molecular structuration, and primary amines exposure, of the self-assembling backbones of CK_3_ and CK_4_.

Hence, different preparations of SAPs were tested to verify if, deviating from the standard SAP handling protocol (i.e., overnight incubation at +4 °C prior usage), was detrimental or beneficial to the overall performance of crossCK_n_. We assessed the outcomes of cross-linking reactions as a result of variations of the incubation times of CK dissolved in water (allowing for the spontaneous formation of loosely interacting nanofibers) [43] and also in relation to different CK concentrations (Supplementary data, Table S1).

In summary, SAP concentration decrements and shortening of the incubation time prior to self-assembling and cross-linking were detrimental to the mechanical properties of crossCK_n_ (see supplementary results) and the standard SAP preparation protocol was confirmed to be the best one. Lastly, even though crossCK_4_ did not place itself at a higher position than crossCK_3_, all crossCK_n_ with 20 mM initial concentration have achieved values of G′ between (170 ± 16) kPa and (532 ± 44) kPa.

### 3.3. ATR-FTIR Experiments

**Figure 4:**
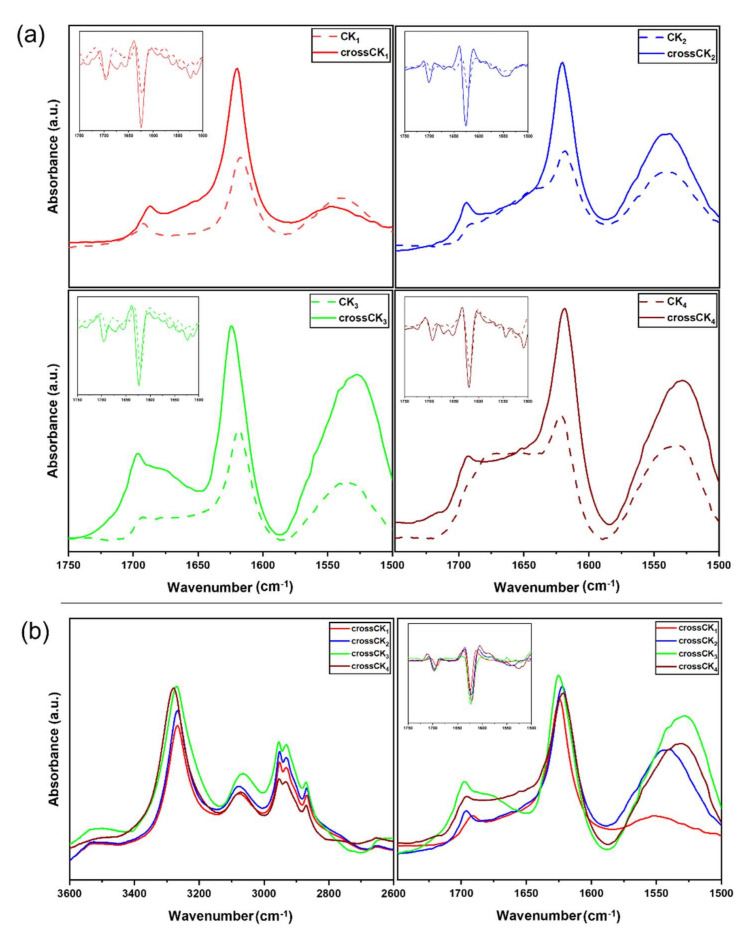
Experimental ATR-FTIR absorption spectra of CK_n_ and crossCK_n_. (**a**) CK_1-4_ and crossCK_1-4_ spectra in the Amide I–II band with their respective second derivatives (inserts). Both CK_1-4_ and crossCK_1-4_ peptides showed typical features of β-sheet structures at ~1620 cm^−1^ and at ~1695 cm^−1^. (**b**) Comparison among crossCK_1-4_ in the Amide A (on the **left**) and Amide I-II regions (on the **right**). All experiments displayed β-sheet structures at ~3300 cm^−1^ and the greatest absorption for crossCK_3_ at ~1695 cm^−1^: all curves showed an increase of β-sheet structuration in the Amide I region once cross-linking occurred.

In ATR-FTIR experiments, infrared radiation absorption spectra were studied in the amide I, II, and A bands. All ATR-FTIR spectra showed the presence of the typical absorption bands of peptides at 3270 cm^−1^, 2930 cm^−1^, 1623 cm^−1^, and 1532 cm^−1^, ascribable respectively to the stretching vibrational bands of -NH_2_, C-H aliphatic, C=O acetyl and to the bending vibrational band of N–H (Figure 4a). Moreover, data confirmed the presence of β-sheet-rich fibers formed after self-assembly [44].

In the Amide I band (Figure 4a), the acetyl group stretching vibration of the peptide bond was observed, demonstrating the sensitivity to the secondary structure and the β-sheet intermolecular interactions of the peptides at 1623 [45].

In the Amide II region, the absorption bands display the bending vibrational bands of the N–H group (as a consequence of the NH_2_ deformation in primary amides) and the N-H bending and C-N stretching vibrations in secondary amides [46]. In the Amide II region, the major contribution came from the strong sharp absorption band at 1532 cm^−1^ ascribable to the C=O stretching. In the cross-linked compounds it was also possible to reveal the shift of the stretching vibrational bands from ~1620 cm^−1^ to ~1625 cm^−1^ because of cross-linking between CK_n_ and Sulfo-SMCC. Furthermore, the Amide A region (Figure 4b) showed peak frequencies typical of a weakly hydrogen bonded NH moiety and a free NH stretch: the peak near ~3300 cm^−1^ indicates the presence of medium strength H-bonds, typical of β-sheet structures.

Mathematical procedures such as Fourier second derivatives and deconvolution were used to resolve the overlapping bands (see Figure S4 in Supplementary Materials). All peptide spectra displayed the presence of intermolecular β-sheet structures with strong H-bonds (1623 cm^−1^) and with high frequency components (1695 cm^−1^). Given the increments in the components ascribable to β-structures found in crossCK_1-3_ if compared to CK_1-3_, it is evident that cross-linking with Sulfo-SMCC favors tighter β-sheets packing of CK_1-3_, likely contributing to the overall increased mechanical properties we previously demonstrated.

On the other hand, amide I peak deconvolution for CK_4_ showed a more heterogeneous composition of secondary structures comprising α-helix, β-sheets, and coils. This observation points out that the increase of the peak intensity between 1647 cm^−1^ and 1700 cm^−1^ is probably due to chain-length dependent aggregated superstructures rather than to β-sheets increments, thus presumably decreasing the order and packing of active sites, found to be crucial for efficient cross-linking [46].

### 3.4. Degree of Cross-Linking by ThT, TNBSA and Elmann’s Assays

**Figure 5:**
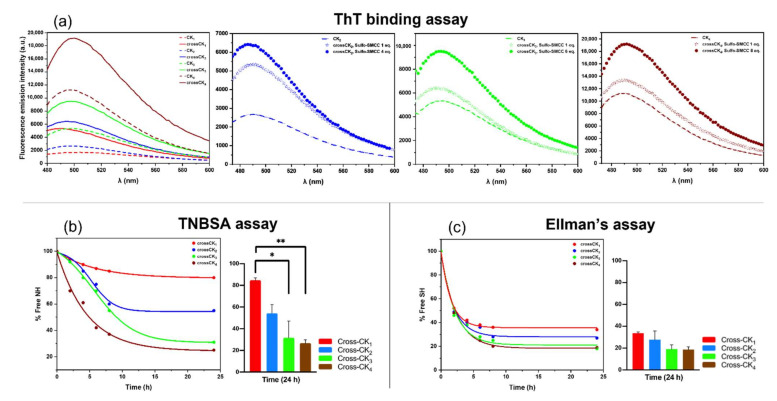
ThT, TNBSA and Ellman’s assays. (**a**) ThT fluorescence emission spectra of CK_1-4_ before and after Sulfo-SMCC cross-linking (Sulfo-SMCC = 2, 4, 6, 8 equivalents for respectively, CK_1_, CK_2_, CK_3_ and CK_4_). To the right, ThT spectra of CK_2-4_ at various concentrations of Sulfo-SMCC: ThT fluorescence levels increased when number of equivalents of Sulfo-SMCC used for the cross-linking reaction matched those of Cys available for binding. (**b**) Absorbance curves from TNBSA assay: all experiments showed a decrease in the free amine percentage during the time course of the reaction: increasing the number of Cys significantly contributed to the decrease of free -NH_2_ groups at the end of the reaction. (**c**) Absorbance curve from Ellman’s assay. All crossCK peptides showed a decrease in free SH-groups during the time course of the reaction until the plateau was reached. In both (**b**,**c**), bars represent the standard deviation of the mean calculated at 24 h where * *p* = 0.0123, ** *p* = 0.0074. Statistical analysis was performed by Ordinary One-way ANOVA.

ThT assay showed fluorescence intensity increments due to β-sheets formation reaching highest values once cross-linking was formed (Figure 5a).

As expected, ThT was shown to bind with favorable affinity to all CKs structures, confirming the presence of β-sheet packed secondary structures in both hydrogels and cross-linked scaffolds. Historically, ThT was used to detect cross-β rich structures only due to the characteristic sigmoidal fluorescence increase occurring from the monomer to the end fibril states. A further optimization was made by adjusting the concentration of Sulfo-SMCC in the cross-linking reaction in accordance with the number of Cys-Gly-Gly motif present in CK_2_, CK_3_ and CK_4_.

Results demonstrated that cross-linking increases the β-structuration of all CK_n_, but also the importance of using the proper equivalents of Sulfo-SMCC matching the number of cysteine residues found in each peptide; indeed, the ThT signal increased along with the concentration of Sulfo-SMCC (Figure 5a).

Furthermore, crossCK_n_ peptides were analyzed by TNBSA assay to indirectly quantify the unmodified free amino groups present in the cross-linked products. As shown in Figure 5b, all crossCK_n_ showed a decreasing percentage of free-NH_2_ immediately after the beginning of the cross-linking reaction, plateauing after 24 h.

Similarly, Ellman’s assay was used to determine the amount of free cysteine/sulfhydryl groups present in the reaction mix [47]. Ellman’s assay values decreased and plateaued with comparable timings to TNBSA, confirming the kinetic of cross-linking reaction and depletion of free SH-groups (Figure 5c). In CK_1_, CK_2_ and CK_3_ the increased numbers of Cys residues allowed for a higher percentage of –NH_2_ to be involved in the cross-linking reaction and was not detrimental to the percentage of remaining free SH-groups. In the case of CK_4_, no significant improvements were detected if compared to CK_3_, both in terms of free -NH_2_ and SH- groups, likely for its more heterogeneous structuration detected in ATR-FTIR data, hampering appropriate exposure and stable “proximity” of active sites to statistically favor cross-links. Apparently, ThT data conflict with β-structuration of CK_4_ detected in ATR-FITR; however, changes in the emission intensity of ThT fluorescence are not univocally attributed [48]. ThT increments were also detected as a consequence of the formation of highly fluorescent dimers [49], excimers [50], and even micelles of ThT itself [51]. Moreover, interactions limiting dye rotation and planarization may also lead to a remarkable increase in fluorescence intensity [52]. Lastly, other studies demonstrated the interference of solvent viscosity on ThT fluorescence behavior [53]. To this purpose, our data suggest that other mechanisms responsible for ThT absorption and fluorescence increments may take place in CK_4_ in addition to standard cross-β-sheet binding, like the probable influence of the long peptidic chain, causing highly-viscous solutions (see rheology section), responsible for unusually high ThT fluorescence.

### 3.5. Morphology

**Figure 6:**
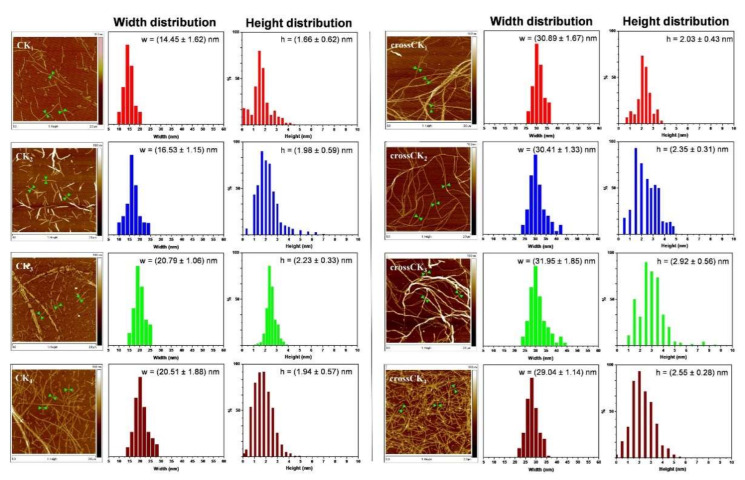
AFM images of CK_n_ peptides (**left**) and crossCK_n_ peptides (**right**) with corresponding height and width distributions. Overall, the cross-linking reaction increased the interactions among nanofibers, yielding to higher and larger bundles of fibers. Height and width profiles were measured using a population of ~50 fibers per each sample. As an example, green arrowheads point at where width profiles were measured.

Nanostructural topography was characterized in both CK_n_ and crossCK_n_. AFM results confirmed that CK_1_, CK_2_, CK_3_ and CK_4_ self-assemble into nanofibers with an average nanofiber width of 15 ± 1.5 nm. On the other hand, crossCK_1_, crossCK_2_, crossCK_3_ and crossCK_4_ featured nanofibers with an average width of 30 ± 1.2 nm, significantly larger than their not cross-linked counterparts. (Figure 6).

This increment is likely given by a higher level of bundling of multiple nanofibers favored by the cross-linking reaction. Interestingly, the gradual increase of fiber width detected from CK_1_ to CK_3_ is easily explained with their increased overall peptide chain length, crucial feature when molecular are perpendicular to the nanofiber axis as in cross-β structures [54,55].

We also characterized the nanofiber height distribution of CK_n_ and crossCK_n_ (Figure 6): AFM analysis revealed similar values among the four uncross-linked SAPs with a height distribution between 1.66 nm and 2.23 nm, while the height distribution for crossCK_n_ laid between 2.03 nm and 2.92 nm, revealing a ~20% height increment after Sulfo-SMCC cross-linking. Nevertheless, neither CK_4_ nor crossCK_4_ exceeded height and width distribution values detected, respectively in CK_3_ and crossCK_3_, seemingly to point out a correlation with the lower mechanical performances of CK_4_ and crossCK_4_. The persistence length (λ) of the detected nanofibers (See Supplementary Methods and Figure S5 in Supplementary Materials) was calculated via the mean-squared midpoint displacement (MSMD) method (characterizing the straightness of nanofibers) and here used as a “geometric” evaluation of their mechanical stiffness [56]. High values of λ suggest that CK_n_ and crossCK_n_ nanofibers can be classified as very stiff fibrillar-like objects (see Supplementary Materials, Figure S5) [57].

### 3.6. Orthogonal Double Cross-Linking

Since crossCK_n_ still featured a residual percentage of free amine groups (TNBSA assay), we aimed at boosting SAP mechanical increments with an additional cross-linking reaction after the first one with Sulfo-SMCC. To this purpose, we chose GP, an aglycone extracted from the iridoid glycoside geniposide present in Gardenia Jasminoides, that can undergo a spontaneous reaction with primary amines present in amino acids [58]. In our previous works, we reported such a cross-linking strategy as being non-cytotoxic for human neural stem cells (hNSC) and of great potential for regenerative medicine applications [6,59].

**Figure 7:**
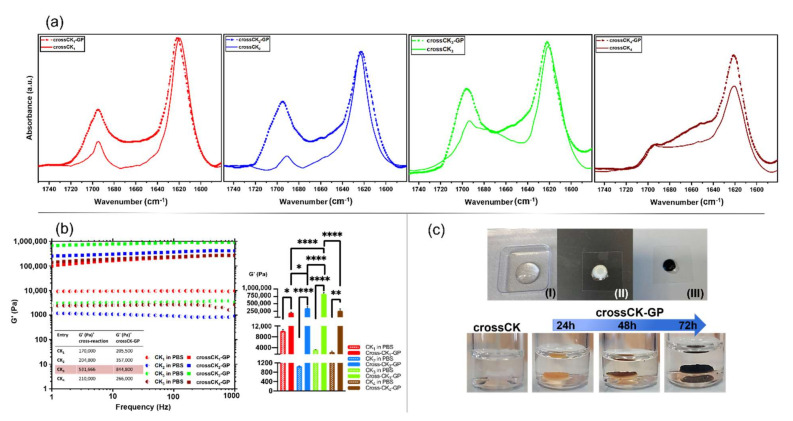
CrossCK_s_-GP experiments. (**a**) Experimental ATR-FTIR absorption spectra of crossCK_n_ and crossCK_n_-GP. (**b**) Rheological tests of crossCK_n_-GP with respective average G′ values: crossCK_3_-GP (highlighted in red) showed the highest value of G′ (844 ± 41) kP. * *p* < 0.05, ** *p* > 0.01 and **** *p* < 0.0001. (**c**) Orthogonal double cross-linking reaction at the macroscale: (**I**) CK_n_ in water solution, (**II**) after self-assembling and Sulfo-SMCC reaction, (**III**) after additional GP cross-linking. Below, imaging of the typical change of color of crossCK_n_-GP over time as a consequence of GP cross-linking.

ATR-FTIR data showed increments of β-sheets component in the Amide I region (peak 1695 cm^−1^) for crossCK_1-3_-GP, but similarly to Sulfo-SMCC cross-linking, not relevant for crossCK_4_-GP (Figure 7a and Figure S6). The absorption peaks of GP after 24 h are shown in Figure S7.

Accordingly, GP cross-linking bestowed crossCK_1-3_ with significantly increased values of G′ reaching (205 ± 20) kPa (crossCK_1_-GP), (357 ± 55) kPa (crossCK_2_-GP), (844 ± 41) kPa (crossCK_3_-GP), but with a limited increment for (crossCK_4_-GP), where it jumped from (208 ± 64) kPa to (266 ± 70) kPa (Figure 7b). Even after GP cross-linking, where all scaffolds turned into a blueish color testifying GP cross-linking (Figure 7c), G″ values were well below the corresponding G′ ones (Figure S6c), testifying the solid behavior of all crossCK_n_-GP scaffolds. Table S3 (Supplementary Materials) compares current results with other cross-linking strategies adopted with SAPs: to the best of our knowledge, cross-CKs and cross-CKs-GP show the highest G’ values obtained with pure SAPs so far.

## 4. Conclusions

Despite the promising potential of SAPs for tissue engineering applications, the usage of such biomimetic versatile scaffolds has been hampered by their poor mechanical properties limiting their applications to fillers or soft tissue replacements [43]. In this work, we designed new SAPs specifically to exploit the Sulfo-SMCC cross-liking approach in terms of stiffer SAP-based nanomaterials. We also demonstrated the feasibility of the orthogonal double cross-linking strategy aimed at bringing G′ values as high as 844 kPa, thus bringing SAP constructs closer to stiffnesses of cardiac tissue, osteoids and skin. Such enhancements came at no cost of the self-assembling propensity of the tested CK_n_: as such they could be applied to a good number of already existing SAPs comprising Lys and Cys in their sequences. Our work demonstrates that multiple selective cross-linking strategies can be taken into account for SAP design, but it also shows some technical limits arising from the unbalanced chain of CK_4_, where long functional motifs hamper appropriate self-assembling and active sites availability for cross-linking. In summary, we laid new theory for the design of cross-linked SAPs, ready to be applied, together with their tuned functionalizations, for the development of stiffer biomimetic scaffolds for the regeneration of different tissues with stiffnesses between ~105 Pa and ~106 Pa, like, for example, including bone tissue, articular cartilage, and tendon connective tissue.

## Data Availability

No public archived data were generated during this study. Data are made available by the authors upon request.

## Supplementary Materials

The following supporting information can be downloaded at: https://www.mdpi.com/article/10.3390/nano12030320/s1, Figure S1: G″ (loss moduli) values; Figure S2: Cross-linking reaction of CK_3_ and CK_4_ at different concentrations and incubation times; Figure S3: Figure S3. G″ (loss moduli) values of CK_3_ and CK_4_ at different concentrations and incubation times; Figure S4. Deconvolution of ATR-FTIR absorption spectra of CK_1-4_ in the Amide I band; Figure S5: Persistence length; Figure S6: CrossCK_n_-GP experiments; Figure S7: ATR-FTIR absorption spectra of Genipin; Table S1: G’ values of cross-linking reaction of CK_3_ and CK_4_ at different concentrations and incubation times. Table S2: G″ values of cross-linking reaction of CK_3_ and CK_4_ at different concentrations and incubation times_._ Table S3. Comparison table of different G’ (storage modulus) obtained in other similar works.
