# Supplementary material for: Boosted Cross-Linking and Characterization of High-Performing Self-Assembling Peptides
Source: Nanomaterials (Basel). 2022 Jan 19;12(3):320. doi: 10.3390/nano12030320 (PMC8838902; doi:10.3390/nano12030320)
Supplement: Supplementary file 1 [file nanomaterials-12-00320-s001.zip › nanomaterials-1546875-supplementary.pdf]

nanomaterials

MDPI

Supplementary Materials

Boosted Cross-Linking and Characterization of High-Performing Self-Assembling Peptides

Maria G. Ciulla [1], Raffaele Pugliese [1,2] and Fabrizio Gelain [1,3,*]

1 Institute for Stem-Cell Biology, Regenerative Medicine and Innovative Therapies, IRCCS Casa Sollievo della Sofferenza, 71013 San Giovanni Rotondo, Italy; mg.ciulla@operapadrepio.it (M.G.C.); raffaele.pugliese@nemolab.it (R.P.)
2 NeMo Lab, ASST Grande Ospedale Metropolitano Niguarda, 20162 Milan, Italy
3 Center for Nanomedicine and Tissue Engineering (CNTE), ASST Grande Ospedale Metropolitano Niguarda, 20162 Milan, Italy
* Correspondence: f.gelain@css-mendel.it; Tel.: +39-02-6444-7519

Citation: Ciulla, M.G.; Pugliese, R.; Gelain, F. Boosted Cross-Linking and Characterization of High-Performing Self-Assembling Peptides. *Nanomaterials* **2022**, *12*, 320. https://doi.org/10.3390/nano12030320

Academic Editor: Miguel Gama

Received: 23 December 2021
Accepted: 16 January 2022
Published: 19 January 2022

Publisher's Note: MDPI stays neutral with regard to jurisdictional claims in published maps and institutional affiliations.

Copyright: © 2022 by the authors. Licensee MDPI, Basel, Switzerland. This article is an open access article distributed under the terms and conditions of the Creative Commons Attribution (CC BY) license (https://creativecommons.org/licenses/by/4.0/).

Supplementary methods

General procedure for the preparation of CKs

All peptides were synthesized *via* microwave assisted Fmoc SPPS on a Rink Amide resin. Coupling conditions were as follows: 4 min, 90 °C, 50 W of microwave energy. The peptide derivative was cleaved from the resin using a cleavage cocktail containing 92.5 % TFA, 2.5 % H_2O, 2.5 % DODt and 2.5 % TIS (v:v:v:v), precipitated in ice cold diethyl ether and lyophilized in CH_3CN/H_2O (25:75). The crude peptide was then purified *via* RP-HPLC.

Estimation of the persistence length

Persistence length (λ) evaluation of the tested SAPs was performed via the mean-squared midpoint displacement (MSMD) method, suited for supposedly stiff one-dimensional objects [1]. MSMD provides a geometric hint of the nanofibers mechanical stiffness[2,3]. The fit is based on the equation describing the nanofiber midpoint deviation along a rod: $u_x^2 = \ell^3/48\lambda$ where u_x^2 is the MSMD between any pair of segments along a tracked-rod contour, separated by an arc length (ℓ). Main assumption is that displacements are small in comparison with the corresponding arc lengths ($u_x \ll \ell$). Fibril flexibility and rigidity can be quantified by comparison of persistence length and internal contour length (L): a fibril is considered flexible when $\lambda \ll L$, rigid when $\lambda \gg L$, and semiflexible when $\lambda \approx L$ [4]. Nanofibers present in this work can be considered as very stiff fibrillar-like objects with persistence length well above the internal contour length. Persistence length was calculated *via FiberApp* software [5].

Peptide quality control tests

CK₁

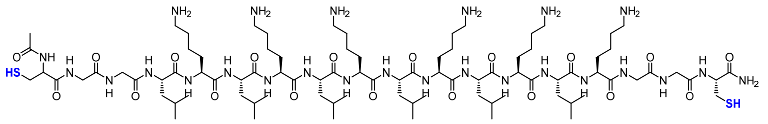

Analytical data:

RP HPLC, C18 column, gradient: 10% → 90% B (CH_3CN + 0.1% TFA), R_t = 18.98 min.

LC-MS (ESI) Calculated for $C_{88}H_{165}N_{25}O_{19}S_2$: m/z, 1940.2155; Found: 1941.2233; $[M+3H]^{+3}$, 647.7463; $[M+4H]^{+4}$, 486.0617.

*CK*2

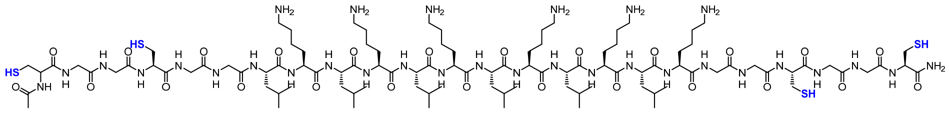

Analytical data:

RP HPLC, C18 column, gradient: 5% → 95% B (CH_3CN + 0.1% TFA), R_t = 7.14 min

LC-MS (ESI) Calculated for $C_{102}H_{187}N_{31}O_{25}S_4$: m/z, 2374.397; Found: $[M+2H]^{+2}$, 1188.1676; $[M+4H]^{+4}$, 594.5877.

*CK*3

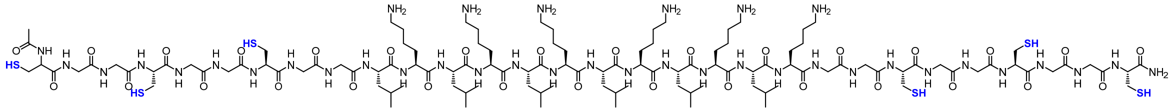

Analytical data:

RP HPLC, C18 column, gradient: 5% → 40% B (CH_3CN + 0.1% TFA), R_t = 14.52 min.

LC-MS (ESI) Calculated for $C_{116}H_{209}N_{37}O_{31}S_6$: m/z, 2808.4239.56; Found: $[M+2H]^{+2}$, 1405.2198; $[M+3H]^{+3}$, 937.1491; $[M+4H]^{+4}$, 703.1138.

*CK*4

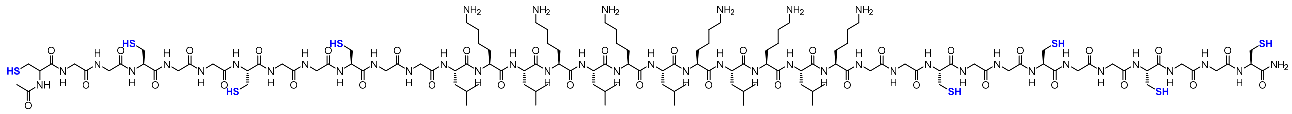

Analytical data:

RP HPLC, C18 column, gradient: 5% → 95% B (CH_3CN + 0.1% TFA), R_t = 9.50 min

Q-TOF-MS-MS Calculated for $C_{88}H_{165}N_{25}O_{19}S_2$: m/z, 3242.56; Found: isotopic peak pattern of $[M+2H]^{+2}$, $[M+3H]^{+3}$ and $[M+4H]^{+4}$.

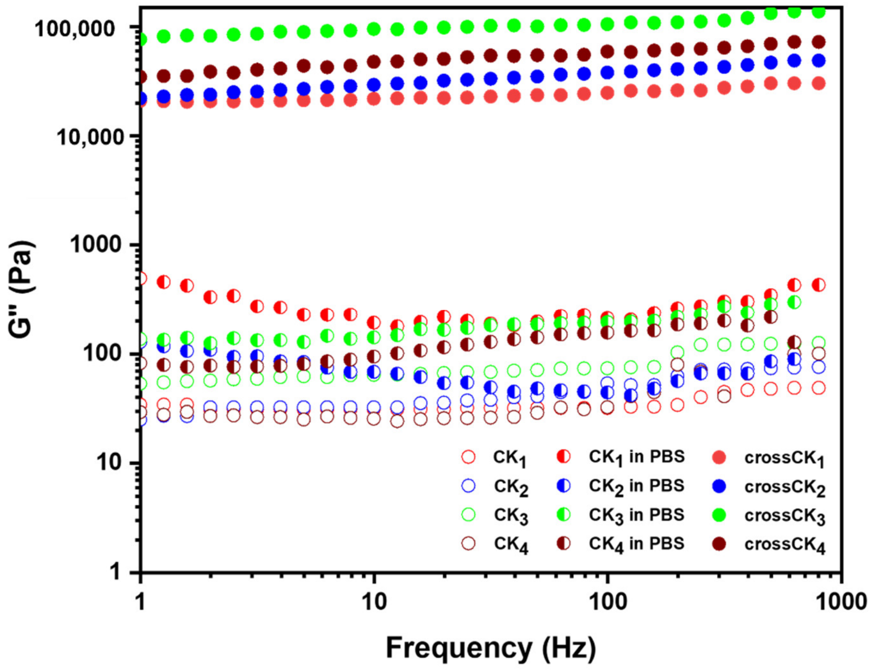

Figure S1. G″ (loss moduli) values. G″ (loss moduli) values were calculated in frequency-sweep tests after self-assembling (PBS) and after Sulfo-SMCC cross-linking reaction. All experiments were performed in triplicate.

Table S1. G′ values of CK_3 and CK_4 after cross-linking reaction at different concentrations and incubation times. Mean values of storage moduli G′ were calculated after incubation, after addition of Dulbecco's phosphate buffer saline solution (DPBS, pH=7.4) and after cross-link reaction via frequency-sweep tests. All experiments were performed in triplicate. In red is highlighted the CK_3 experimental condition giving the highest G′ value after cross-linking: CK_3 showed the highest G′ value in the case of 20 mM initial concentration and after an overnight incubation at +4°C. Indeed, G′ of crossCK_3 when [CK_3] 20 mM > crossCK_3 when [CK_3] was 15 mM > crossCK_3 when [CK_3] 10 mM. On the other hand, G′ of crossCK_4 when [CK_4] 20 mM > crossCK_4 when [CK_4] was 15 mM > crossCK_4 when [CK_4] 10 mM (see also figure S2).

Entry	Peptide	[CK] mM	pre-assembly incubation-time	G' (Pa)*	G' (Pa)* in DPBS	G' (Pa)* cross-reaction
1	CK_3	10 mM	ov. at + 4°C	188	5200	46,100
2	CK_3	15 mM	ov. at + 4°C	27	5649	84,310
3	CK_3	20 mM	ov. at + 4°C	165	3217	531,666
4	CK_3	20 mM	1h at + 4°C	599	3152	340,000
5	CK_4	10 mM	ov. at + 4°C	72	1514	8840
6	CK_4	15 mM	ov. at + 4°C	413	4200	72,000
7	CK_4	20 mM	ov. at + 4°C	2364	2462	210,000
8	CK_4	15 mM	1h at + 4°C	68	1342	72,070
9	CK_4	20 mM	1h at + 4°C	289	1120	49,940

Table S2. G″ values of CK_3 and CK_4 after cross-linking reaction at different concentrations and incubation times. Main values of loss moduli G″ (Pa) monitored after incubation, after addition of Dulbecco's phosphate buffer saline solution (DPBS, pH=7.4) and after cross-linking reaction.

Entry	Peptide	[CK] mM	pre-assembly incubation-time	G'' (Pa)*	G'' (Pa)* in DPBS	G'' (Pa)* cross-reaction
1	CK_3	10 mM	ov. at + 4°C	7	339	6096
2	CK_3	15 mM	ov. at + 4°C	6	319	16,520
3	CK_3	20 mM	ov. at + 4°C	27	158	94,000

4	CK_3	20 mM	1h at + 4°C	35	185	53,850
5	CK_4	10 mM	ov. at + 4°C	4	180	1591
6	CK_4	15 mM	ov. at + 4°C	35	178	14,220
7	CK_4	20 mM	ov. at + 4°C	27	106	46,950
8	CK_4	15 mM	1h at + 4°C	9	108	14,250
9	CK_4	20 mM	1h at + 4°C	51	75	14,060

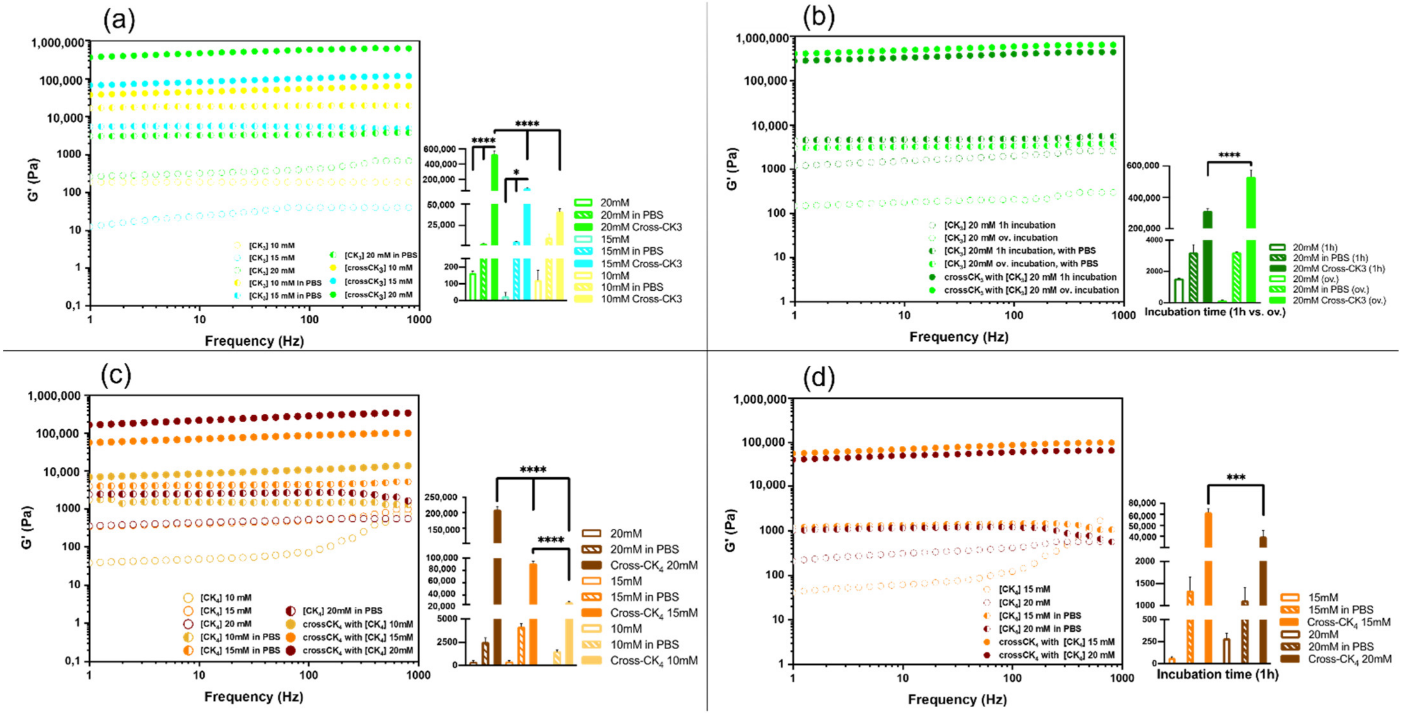

Figure S2. Cross-linking reaction of CK_3 and CK_4 at different concentrations and incubation times. (a) Frequency sweep tests for crossCK_3 at different concentration of CK_3, 10 mM (yellow), 15 mM (light blu) and 20 mM (green). (b) Frequency sweep tests for crossCK_3 at different incubation times (20mM CK_3), 1 hour (dark green), overnight (green). (c) Frequency sweep tests for crossCK_4, at different concentration of CK_4, 10 mM (sand), 15 mM (orange) and 20 mM (brown). (d) G′ comparison of crossCK_4 at 1 h incubation time for concentration of CK_4 15 mM (orange) and CK_4 20 mM (brown). All experiments were performed in triplicate. Statistical analysis was performed via Ordinary One-way ANOVA. No appreciable improvements were observed by shortening the incubation time, i.e. when the SAPs were dissolved and incubated for 1 hour at +4°C instead of 12 hours at +4°C. *$p<0.004$, ***$p<0.001$, ****$p<0.0001$.

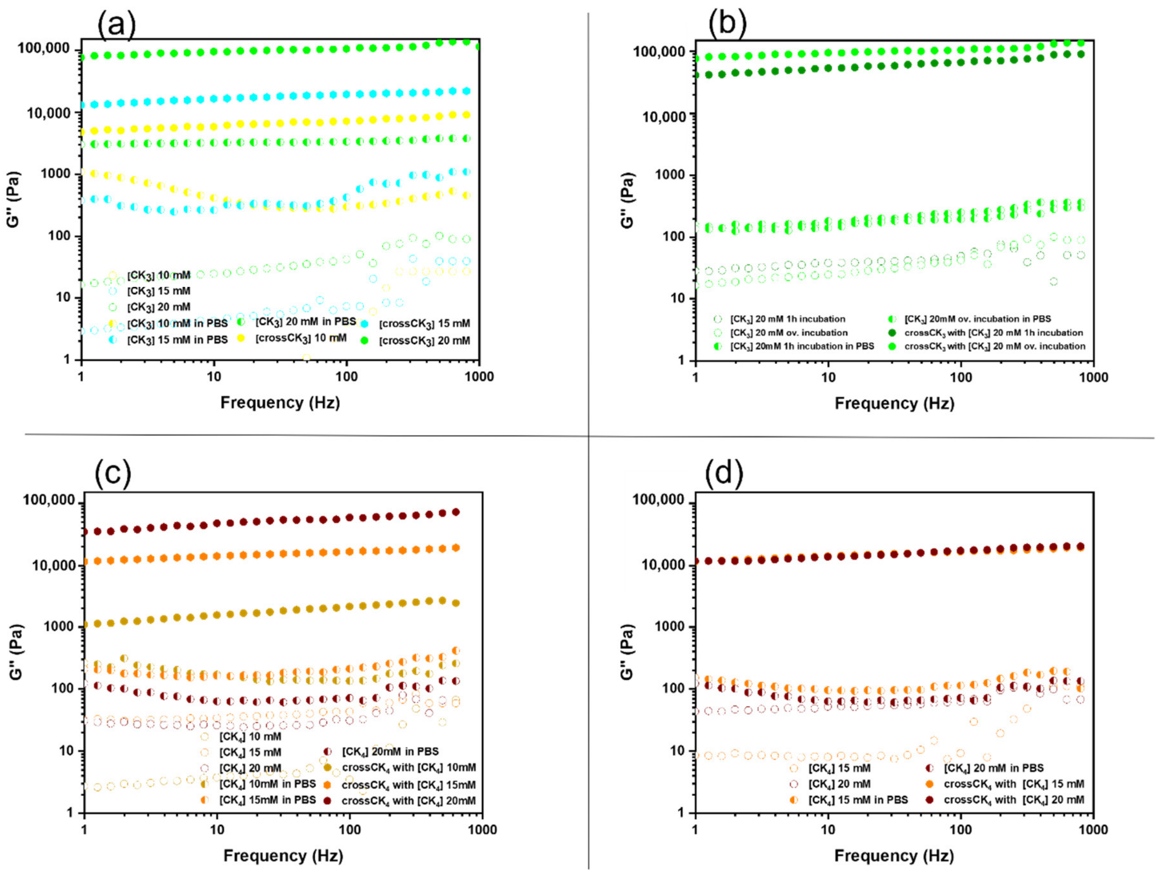

Figure S3. G″ (loss moduli) values of CK_3 and CK_4 at different concentrations and incubation times. Values were obtained after Sulfo-SMCC cross-link reaction *via* frequency-sweep tests. All experiments were performed in triplicate.

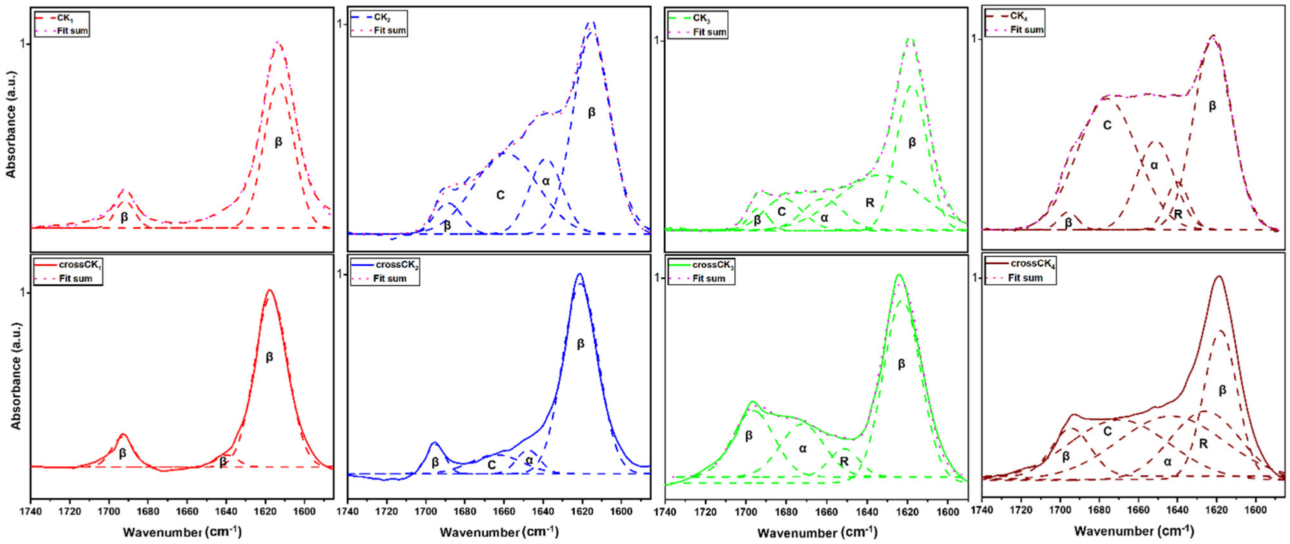

Figure S4. Deconvolution of ATR-FTIR absorption spectra of $CK_{1\text{-}4}$ in the Amide I band. After baseline correction, hidden peaks were detected by using the second derivative method followed by smoothing with the 7–9 point Savitsky–Golay function

(polynomial order=2). Peak fitting/deconvolution was performed using the Voigt function (OriginPro), which is the convolution of a Gaussian function and a Lorentzian function [6]. α: α-helix; β: β-sheets; C: coils; R: random.

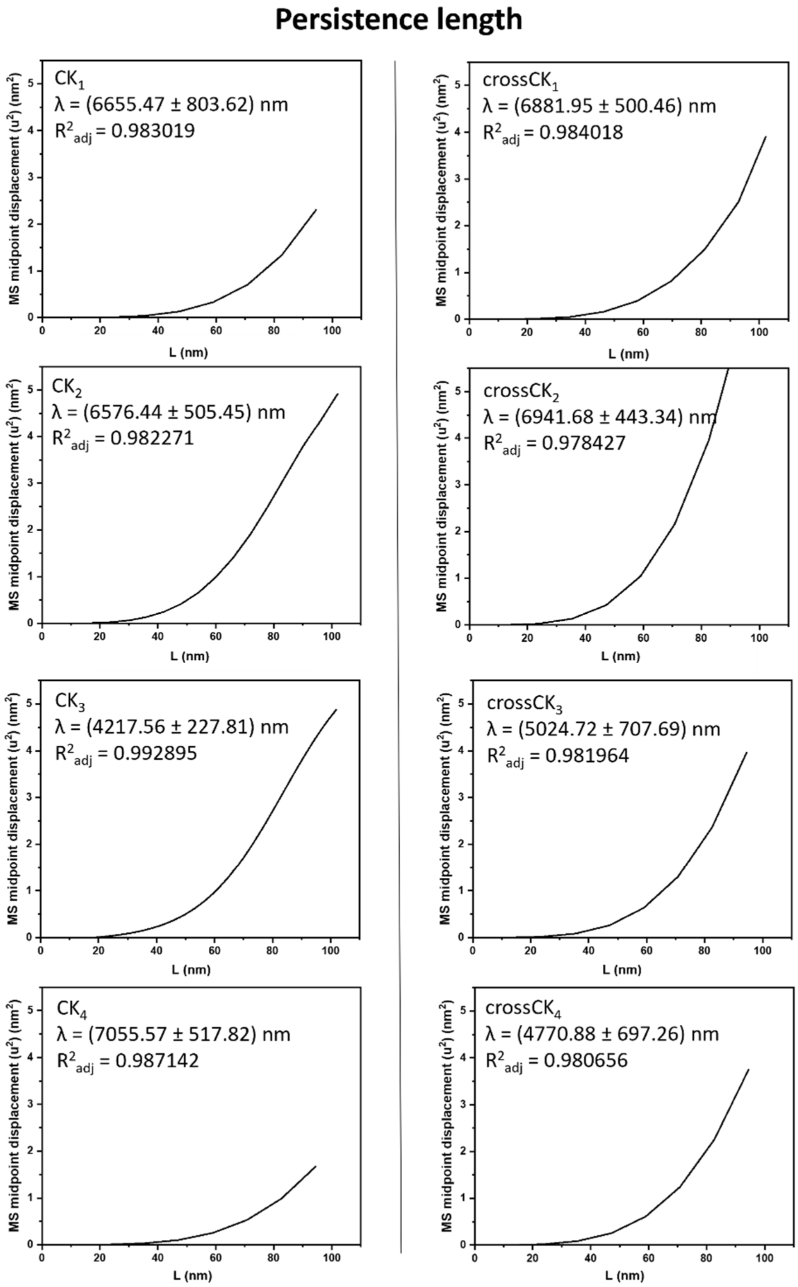

Figure S5. Persistence length. Mean persistence length (λ), calculated *via* mean-squared midpoint displacement (MSMD) method (with L ranging from 0 to 100 nm). Since $\lambda \gg$ L the detected nanostructures could be considered as stiff nanofibrillar objects in all CK_n and $crossCK_n$. R^2 = mean square deviation.

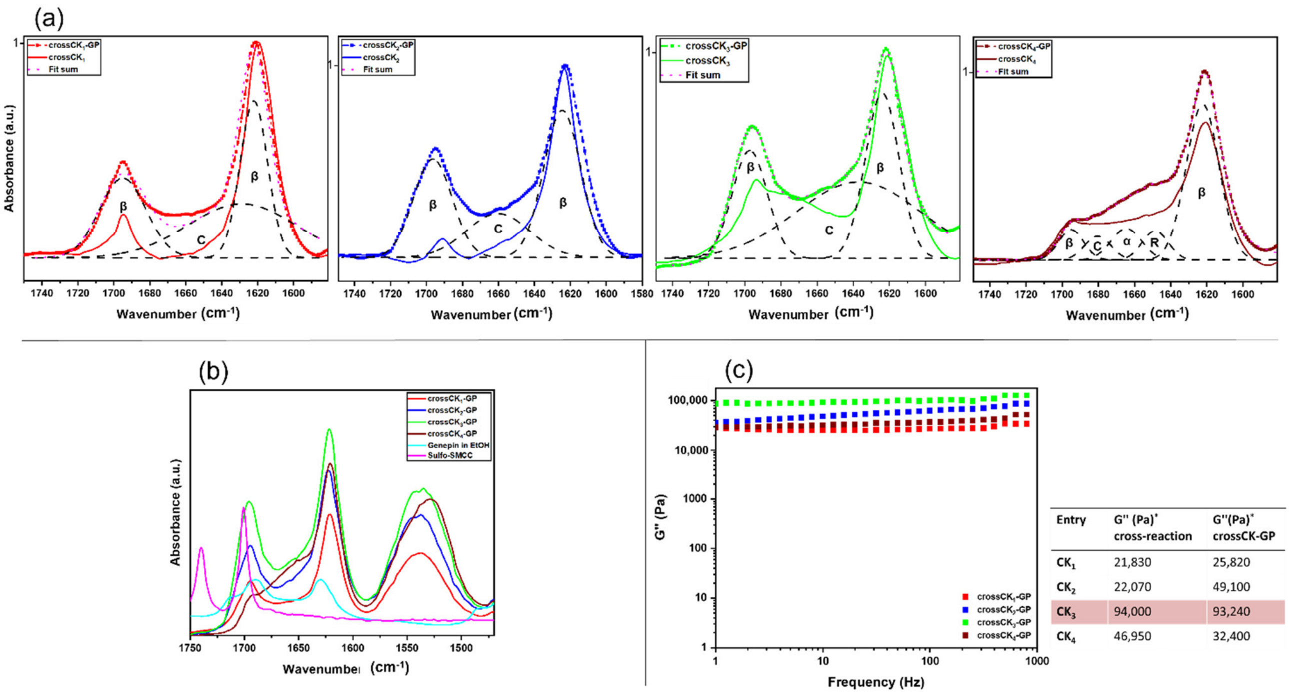

Figure S6. CrossCKn-GP experiments. (a) Deconvolution of ATR-FTIR absorption spectra in the Amide I region for crossCKn and crossCKn-GP. α: α-helix; β: β-sheets; C: coils; R: random. (b) Superimposed ATR-FTIR absorption spectra (Amide I region) of CrossCKn-GP. (c) G″ (loss moduli) values calculated after double cross-linking with Sulfo-SMCC and Genipin.

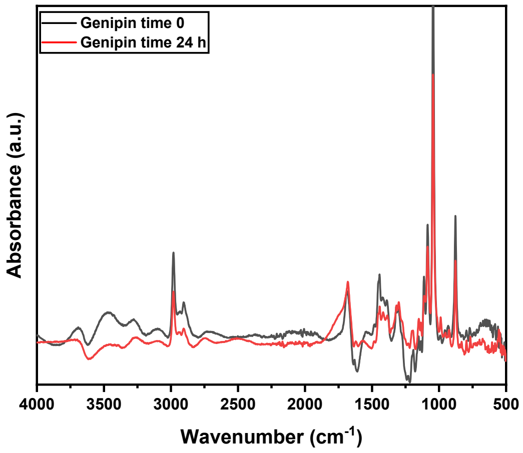

Figure S7. ATR-FTIR absorption spectra of Genipin before and after cross-linking reaction (24 hours).

Table S3. Comparison table of G′ values of various SAPs cross-linked with different compounds. Entries 1-3 (pink background) are referred to this work.

Entry	Reactant	Cross-linker	G' (kPa)	REF.
1	CK_3	Sulfo-SMCC+Genipin	~ 844	
2	CK_1	Sulfo-SMCC	~ 170	
3	CK_1	Sulfo-SMCC+Genipin	~ 205	
4	Lauryl-VVAGKK-Am	Glutaraldehyde	~ 80	[7]
5	$FAQ(LDLK)_3$	Genipin	~ 80	[8]
6	$(LDLK)_3$	EDC-NHS	~ 2,2	[9]
7	$FAQ(LDLK)_3$	Guar Guam	~ 2 to ~ 60	[10]

8	$(LKLK)_3$	$SM(PEG)_{24}$	~ 5 to 60	[11]
9	$(LDLK)_3$	Rubpy	~ 26	[12]

References

1. Dongdong, W.; Nairiti, S.; Jeeyoung, L.; et al. Polymers with controlled assembly and rigidity made with click-functional peptide bundles. *Nature*. **2019**, *574*(7780), 658-662.

2. Gittes, F.; Mickey, B.; Nettleton, J.; Howard, J. Flexural rigidity of microtubules and actin filaments measured from thermal fluctuations in shape. *J Cell Biol*. **1993**, 120(4), 923-934.

3. Broedersz, CP. Modeling semiflexible polymer networks. *Rev Mod Phys*. **2014**, *86*, 995.

4. Cao, Y.; Bolisetty, S.; Adamcik, J.; Mezzenga, R. Elasticity in Physically Cross-Linked Amyloid Fibril Networks. *Phys Rev Lett*. **2018**, *120*(15), 158103.

5. Usov, I.; Mezzenga, R. FiberApp: An Open-Source Software for Tracking and Analyzing Polymers, Filaments, Biomacromolecules, and Fibrous Objects. *Macromol*. **2015**, *48*(5), 1269-1280.

6. Ji, Y.; Yang, X.; Ji, Z.; Linhui, Z.; Nana M.; Dejun, C.; Xianbin, J.; Junming, T.; Yilin, C. DFT-Calculated IR Spectrum Amide I, II, and III Band Contributions of N-Methylacetamide Fine Components. *ACS Omega*. **2020**, *5*, 8572-8578.

7. Khalily, M.A.; Goktas, M.; Guler, M.O. Tuning viscoelastic properties of supramolecular peptide gels via dynamic covalent crosslinking. *Org. Biomol. Chem.* **2015**, *13*(7), 1983–1987.

8. Pugliese, R.; Maleki, M.; Zuckermann, R. N.; Gelain, F. Self-assembling peptides cross-linked with Genipin: resilient hydrogels and self-standing electrospun scaffolds for tissue engineering applications. *Biomater. Sci*. **2019**, *7*, 76-91.

9. Pugliese, R.; Gelain, F. Cross-Linked Self-Assembling Peptides and Their Post-Assembly Functionalization via One-Pot and In Situ Gelation System. *Int. J. Mol. Sci.* **2020**, *21*, 4261.

10. Pugliese, R.; Gelain, F. Characterization of elastic, thermo-responsive, self-healable supramolecular hydrogel made of self-assembly peptides and guar gum. *Mater. Des*. **2020**, *186*, 108370.

11. Pugliese, R.; Gelain, F. Programmable stiffness and stress–relaxation of cross-linked self-assembling peptide hydrogels. *J Appl Polym Sci*. **2021**, e51759.

12. Pugliese, R.; Montuori, M.; Gelain, F. Bioinspired photo-crosslinkable self-assembling peptides with pH-switchable "on–off" luminescence. *Nanoscale Adv*.**, 2022**, Advance Article.
